# Personal

**Published:** 1904-01

**Authors:** 


### PERSONAL.

Dr. Edward F. Brush, of Mount Vernon, N. Y., was elected mayor of that city at the general election held in November, 1903. He has held before the same office and during his term of service rescued the city from threatened bankruptcy. It is stated that a similar opportunity presents itself to him at the threshold of his present term. Dr. Brush is well known as one of the most prominent physicians in the Westchester district.

Dr. Eugene Beach, of Gloversville, was elected mayor of that city, November 3, 1903. Dr. Beach is also a state medical examiner, but has never before held political office. He will have an opportunity to institute needed reforms during his term of office.

Dr. Willis F. Westmoreland, of Atlanta, has been appointed president of the Georgia state board of health, a body created by recent legislative enactment. Dr. Westmoreland will, therefore, have ample opportunity for the exercise of his well-known executive ability in establishing sanitary regulations throughout his native state.

Dr. George H. Westinghouse, of Buffalo, has been appointed district physician, vice Eugene C. Waldruff, removed. Dr. Westinghouse will exchange districts with Dr. De Groat, for the sake of convenience. The appointment of Dr. Westinghouse cannot fail to strengthen the hands of the Commissioner of Health, as he thereby secures a distinct and creditable addition to his staff.

Dr. Charles H. B. Meade, formerly of Buffalo, has been appointed surgeon in the Cunard steamship service. He sailed from New York on the Carpathia, November 24, 1903, for a cruise in the Mediterranean.

Dr. Roswell Park, of Buffalo, delivered an address Friday evening, November 27, 1903, before the Buffalo Society of Natural Science, choosing for his subject, Radium and radioactive substances.

Dr. William C. Krauss, of Buffalo, addressed the Society of Natural Sciences, Friday evening, December 11, 1903, on the Height and weight of normal and abnormal individuals.

Dr. Charles Haase, a graduate of the University of Buffalo, 1902, and who afterward served a term as interne at the Sisters of Charity Hospital, which he completed November 1, 1903, has located at 17 Walden Avenue, corner of Genesee Street, Buffalo. Hours: 8 to 9 a. m.; 12 to 2 and 6 to 8 p. m. Telephone, Howard 2543.

---

Dr. Ray H. Johnson, of Buffalo, has removed his residence and home office to 449 Franklin Street. Hours: 1.30 to 2.30 p. m. He still retains a branch office at his former residence, corner of North Division and Chestnut Streets. Hours: 12 m. and 7 p. m. Telephones: Bell, Tupper 850; Frontier, 850. At branch office, Frontier, 26072.

---

Dr. Frederick H. Millener, of Buffalo, addressed the Historical Society, November 22, 1903, on the history of the telephone.

---

Dr. H. G. Matzinger, of Buffalo, lectured on artistic anatomy before the Art Students' League, December 16, 1903.

---

Hon. T. Guilford Smith, of Buffalo, a regent of the University of the State of New York, was elected a member of the executive committee of the board of regents for 1904, at the annual meeting of that body held in December. Regent Smith was also appointed a member of the committees on finance and museum, being chairman of the former committee.

---

Rev. Arthur E. Mann, son of Dr. Matthew D. Mann, of Buffalo, who was ordained recently by Bishop Walker of the diocese of Western New York, has been appointed a teacher in St. John's College, Shanghai, China. Mr. Mann sailed for his new post of duty December 28, 1903.

